# *Withania somnifera* (L.) Dunal: Opportunity for Clinical Repurposing in COVID-19 Management

**DOI:** 10.3389/fphar.2021.623795

**Published:** 2021-05-03

**Authors:** Akash Saggam, Kirti Limgaokar, Swapnil Borse, Preeti Chavan-Gautam, Santosh Dixit, Girish Tillu, Bhushan Patwardhan

**Affiliations:** ^1^ AYUSH Center of Excellence, Center for Complementary and Integrative Health, Interdisciplinary School of Health Sciences, Savitribai Phule Pune University, Pune, India; ^2^ Division of Biochemistry, Department of Chemistry, Fergusson College (Autonomous), Pune, India; ^3^ Prashanti Cancer Care Mission, Pune, India

**Keywords:** Ashwagandha, Ayurveda, *Rasayana*, Immunomodulation, Inflammation, Cytokine, Adjuvant

## Abstract

As the COVID-19 pandemic is progressing, the therapeutic gaps in conventional management have highlighted the need for the integration of traditional knowledge systems with modern medicine. Ayurvedic medicines, especially Ashwagandha (*Withania somnifera* (L.) Dunal, WS), may be beneficial in the management of COVID-19. WS is a widely prescribed Ayurvedic botanical known as an immunomodulatory, antiviral, anti-inflammatory, and adaptogenic agent. The chemical profile and pharmacological activities of WS have been extensively reported. Several clinical studies have reported its safety for use in humans. This review presents a research synthesis of *in silico*, *in vitro*, *in vivo*, and clinical studies on *Withania somnifera* (L.) Dunal (WS) and discusses its potential for prophylaxis and management of COVID-19. We have collated the data from studies on WS that focused on viral infections (HIV, HSV, H1N1 influenza, etc.) and noncommunicable diseases (hypertension, diabetes, cancer, etc.). The experimental literature indicates that WS has the potential for 1) maintaining immune homeostasis, 2) regulating inflammation, 3) suppressing pro-inflammatory cytokines, 4) organ protection (nervous system, heart, lung, liver, and kidney), and 5) anti-stress, antihypertensive, and antidiabetic activities. Using these trends, the review presents a triangulation of Ayurveda wisdom, pharmacological properties, and COVID-19 pathophysiology ranging from viral entry to end-stage acute respiratory distress syndrome (ARDS). The review proposes WS as a potential therapeutic adjuvant for various stages of COVID-19 management. WS may also have beneficial effects on comorbidities associated with the COVID-19. However, systematic studies are needed to realize the potential of WS for improving clinical outcome of patients with COVID-19.

## Introduction

The COVID-19 or coronavirus disease 2019 is a contagious disease caused by SARS-CoV-2. The rapidly spreading disease is considered as one of the causes of mortality globally (Zhou P. et al., 2020) (“WHO Announces COVID-19 Outbreak a Pandemic” 2020) (“WHO Coronavirus Disease (COVID-19) Dashboard” 2020).

Understanding the pathophysiology of this disease is rapidly advancing with the availability of new research data. Current evidence suggests that most individuals are asymptomatic or are suffering from mild symptoms. The patients who progress to severity develop pneumonia and ARDS and require hospitalization. The SARS-CoV-2 virus uses the angiotensin-converting enzyme 2 (ACE2) receptor for cell entry and the transmembrane serine protease 2 (TMPRSS2) for spike protein priming (Hoffmann et al., 2020). Upon host cell entry, +ssRNA is released which then takes over the cellular ribosomal machinery to synthesize structural proteins and enzymes essential for viral replication (Du et al., 2009) (Alanagreh et al., 2020). The infected cells recruit immune cells for viral clearance, which release cytokines combatively that induce hyperinflammation leading to organ damage (Mortaz et al., 2020). Thus, COVID-19 is characterized by collapsed immune balance, hyperinflammation, cytokine storm, and multiorgan failure. The common clinical symptoms of COVID-19 observed so far are fever, cough, breathlessness, and fatigue. The pathological observations revealed ground-glass opacities, pneumonia, and hematological abnormalities (Fu et al., 2020). Patients with comorbidities such as diabetes, hypertension, cancer, tuberculosis, etc., are at an increased risk of complications (Sanyaolu et al., 2020).

The extent of COVID-19 has highlighted challenges for healthcare systems. The advancing clinical observations are rapidly changing the management protocols. The present empirical pharmacotherapeutics involves antivirals (e.g., remdesivir), antimalarials (e.g., chloroquine and hydroxychloroquine), antibiotics (e.g., azithromycin), and in few cases immunomodulators (e.g., tocilizumab) (“Clinical Management of Severe Acute Respiratory Infection When COVID-19 Is Suspected” 2020) (“Revised Advisory on the Use of Hydroxychloroquine (HCQ) as Prophylaxis for SARS-CoV-2 Infection” 2020) (“Clinical Management Protocol”, 2020). However, the adverse effects of these drugs remained a concern. An approach of convalescent plasma therapy is also being explored (Rajendran et al., 2020). The accelerated vaccine development has undergone clinical trials and obtained the provisional license for emergency use in a pandemic. The rapid outbreak of the disease necessitates an integration of traditional knowledge systems with modern medicine. Ayurveda, an ancient Indian medicine system, can provide probable candidates for natural product drug discovery even for emerging diseases (Patwardhan et al., 2004). The traditional wisdom of Ayurvedic medicine has a lot to offer for the management of COVID-19.

*Withania somnifera* (L.) Dunal (Ashwagandha/WS) is one of the extensively prescribed botanicals in Ayurveda practice for its multimodal effects (Vaidya, 2000). The diverse pharmacological activities including immunomodulatory, anti-inflammatory, antioxidant, anti-stress, antihypertensive, and antidiabetic along with organ-protective effects have been studied extensively by researchers (Mishra et al., 2000). The scientific evidence supports the prophylactic effect of WS to maintain immune homeostasis in inflammatory and infectious diseases (Minhas et al., 2011) (Teixeira et al., 2006).

The chemical profile of several extracts and formulations of WS has been well documented in previous studies. Briefly, withanolides (steroidal lactones), the main phytochemical of WS, play a central role in exhibiting multimodal effects synergistically. These are a group of C_28_-steroidal lactone triterpenoids, which majorly include withaferin A, withanolide A, B, and D, withanoside IV and V, withasomniferin A, withanone, sitoindoside IX and X, 12-deoxywithastramonolide, etc. Moreover, other polyphenols including catechin, naringenin, syringic acid, and p-coumaric acid were also found in significant quantities in WS extracts. A combination of such versatile phytochemicals potentiates WS as a strong therapeutic agent (Kalra and Kaushik, 2017) (Alam et al., 2011).

This is a narrative review based on reported scientific literature of experimental studies preferably indexed in PubMed database. The collected properties are represented in compliance with traditional use of WS as per Ayurveda literature. Using the search terms such as immunity, cytokine modulation, inflammation, and organ protection, the review analyses the literature for several pharmacological activities of WS. These search terms were chosen in the context of pathophysiological aspects of COVID-19.

This review collates the biochemical actions of WS on several viral infections and diseases based on available literature. These actions are mapped, with the background of advancing pathophysiological insights of COVID-19. Depending upon the available scientific evidence, the review advocates WS as an adjuvant to current pharmacotherapeutics, underlining an integrative approach in COVID-19. The review also suggests use of WS in the management of comorbidities. Collectively, the review presents a research synthesis of reported *in silico*, *in vitro*, *in vivo*, and clinical studies that offer therapeutic benefits of WS for prophylaxis and clinical management of COVID-19.

## Probable Role of *Withania somnifera* in COVID-19 Pathophysiology

**FIGURE 1:**
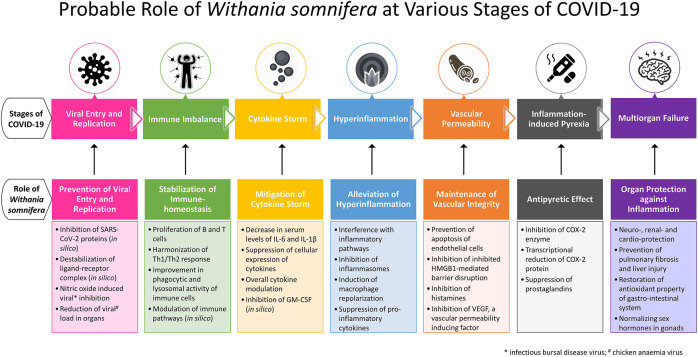
Probable role of *Withania somnifera* at various stages of COVID-19.

Especially in symptomatic patients, COVID-19 exhibits pathophysiological milestones such as viral entry followed by a variety of clinical manifestations. A few patients progress to immune response with cytokine storm and hyperinflammation followed by multi-organ failure. WS is reported to mitigate prior pathophysiological aspects in disease progression and protect vital organs (Figure 1). This section of the review maps the pharmacological properties supported by molecular mechanisms of WS to pathophysiological milestones of COVID-19.

### Viral Entry and Load

SARS-CoV-2 preferentially attacks pneumocytes for their multiplication. This occurs by the binding of viral spike protein to cellular ACE2 receptor followed by viral endocytosis (Du et al., 2009). The virus utilizes ribosomal machinery for mRNA translation into viral proteins with simultaneous mRNA replication using RNA-dependent RNA polymerase (RdRp) enzyme. The viral copies are exocytosed out of infected cells for further encroachments (Alanagreh et al., 2020).

The antiviral properties of WS may interfere with viral entry and subsequent life cycle. The metabolites of WS were explored for their antiviral potential against SARS-CoV-2 proteins using a computational molecular docking tool. It is indicated that withanone may disrupt host–virus interaction by destabilizing the complex of ACE2 and receptor-binding domain of spike protein (Balkrishna et al., 2021). Withaferin A and withanone are predicted to block main protease (Mpro) and TMPRSS2 enzyme to interfere with viral entry and replication (Kumar et al., 2020) (“Ashwagandha Takes Lead in IIT-Delhi Study to Be COVID-19 Warrior”, 2020) (Sharma. S. and Deep, 2020). This was also supported by an *in vitro* study that revealed withanone to downregulate mRNA of TMPRSS2 in MCF7 cells (Kumar et al., 2020). A study carried out by our team predicted the potential of withacoagin and withanolide B to block viral spike protein and RdRp enzyme with a high binding affinity (Borse et al., 2020). Moreover, many recent virtual screening studies reported the potential of withanolides to inhibit SARS-CoV-2 proteins with a high binding affinity (Khanal et al., 2021) (Srivastava et al., 2020) (Parida et al., 2020) (Shree et al., 2020) (Tripathi. M. K. et al., 2020) (Chikhale et al., 2020). One of the molecular docking analysis revealed that withaferin A inhibited host receptor glucose-regulated protein 78 (GRP78) found to be upregulated in patients with COVID-19 (Sudeep et al., 2020) (Sabirli et al., 2021). The pharmacophore of withanolides is found to be associated with the induction of cytoprotective heat-shock response (Wijeratne et al., 2014) and inhibition of HSP90 (Gu et al., 2014). A recent report shows the dependency of SARS-CoV-2 on host HSP90 (Li C. et al., 2020) (Kasperkiewicz, 2021). Therefore, detailed pharmaco-mechanistic studies may help to decipher the potential of withanolides in SARS-CoV-2 viral inhibition.

The overarching potential of WS in several other viral diseases is quite evident. A molecular docking and simulation study revealed a high binding affinity of withaferin A with neuraminidase of H1N1 influenza virus (Cai et al., 2015) and DNA polymerase of herpes simplex virus (Grover et al., 2011) at the functional site of enzymes. Administration of WS extract (1%) through feed reduced viral load of lymphoid organs such as thymus and spleen in anemia virus-infected chicks (Latheef et al., 2017). Chick models of infectious bursal disease (IBD) also showed a reduced viral persistence accompanied with lymphocyte stimulation on feeding with a dietary supplement of 1% root powder of WS (Ganguly et al., 2020). The same group of researchers predicted stimulation of NO production by WS extract to inhibit the IBD virus *in vitro* (Ganguly et al., 2018). An *ex vivo* experiment carried out on PBMCs of HIV patients suggested that WS aqueous extract reduces the expression of disease progression marker CD38 on CD8^+^ T lymphocytes. This necessitates the probable anti-HIV potential of WS (Maurya et al., 2019).

A recent overview of *in vitro* studies indicated the importance of polyphenols against SARS-CoV-2 infection (Annunziata et al., 2020). A group of catechin derivatives screened against SARS-CoV nucleocapsid protein showed a significant inhibitory effect (Roh, 2012). Therefore, the potential of methanolic extract of WS containing high polyphenol concentrations (catechin, naringenin, syringic acid, p-coumaric acid, etc.) to inhibit SARS-CoV-2 infection is scientifically intuitive (Alam et al., 2011).

Computational approaches also suggest possible molecular mechanisms regarding the ability of WS to inhibit viral entry. However, experimental and clinical data are limited. This highlights the need for the investigation of WS metabolites as antiviral agents in COVID-19 with systematic experiments.

### Immune Homeostasis

An infectious agent triggers a pathological immune response orchestrated by the several types of immune cells along with secreted biomolecules. An immediate immune response to infections includes antigen recognition and phagocytosis. It is followed by the delayed response of inflammation (Tosi, 2005). In SARS-CoV-2 infection, a sudden elevation of immune response brings the biological architecture at high risk due to hypersecretion of inflammatory molecules (Tufan et al., 2020). This infection also triggers gross pathological alterations in immune cells leading to immune imbalance (Gustine and Jones, 2020). Under such circumstances an intervention is needed, which can balance the immune response in a way that is host tolerable and adverse to viral replication. Interestingly, the collected scientific reports on immunomodulatory effects indicate that WS maintains immune homeostasis rather than unidirectional stimulation or suppression (Patwardhan et al., 2020).

An observational clinical study on isolated PBMCs of patients with COVID-19 showed elevated innate immunity (macrophages and neutrophils) and lowered cell-mediated immunity (B and T cells) (Sadanandam et al., 2020). The T-cell response is highlighted to protect against viral infections including COVID-19 (Luo et al., 2020). An aqueous and hydroalcoholic extract of WS and withanolide A significantly improved cell-mediated immune response. It promoted proliferation of B and T cells along with Th1 response in healthy, chronically stressed, ovalbumin antigen immunized and immunocompromised mice (Khan. B.et al., 2006) (Malik et al., 2007) (Kour et al., 2009) (Khan. S. et al., 2009) (Bani et al., 2006; Sun, 2019). Different chemotypes of WS hydroethanolic extract significantly modulated the proliferation of B and T cells with special reference to balanced Th1/Th2 response in healthy mice (Susheela Kushwaha et al., 2012a). WS hydromethanolic and aqueous root extract administered in healthy mice showed enhanced leukocyte and platelet counts (Davis and Kuttan, 2000) (Agarwal et al., 1999). The immunomodulatory potential of WS extract in viral diseases was also tested in chicks. WS depicted significant cell-mediated immune response confirmed by an increased count of CD4^+^ and CD8^+^ T cells compared with control (Latheef et al., 2017) (Davis and Kuttan, 2002). Similarly, aqueous and ethanolic extract of WS roots and leaves normalized the leukocyte count in *E. coli* infected guinea pigs that were proportionate to control (El-Boshy et al., 2013). Additionally, several fractions of WS hydroethanolic extract and withaferin A significantly modulated the Th1/Th2 response in parasite-infected mouse model (Kushwaha S. et al., 2012b). Dietary supplementation of WS root powder to Rohu fishes improved the phagocytic and lysosomal activity of immune cells over a month. This was accompanied by a significant increase in the survival rate of fishes even after bacterial infection (Arun Sharma et al., 2010). Phagocytic activity of peritoneal macrophages was also enhanced in healthy mice administered with WS hydromethanolic extract (Davis and Kuttan, 2000). An *in silico* network ethnopharmacological investigation by our group revealed the ability of bioactive compounds of WS to modulate immune pathways (Chandran and Patwardhan, 2016). However, it needs to be combined with experimental pharmacology using suitable models. A clinical study revealed an immune-activating effect of the WS root extract administered with *anupana* (i.e., whole milk as a vehicle) as was indicated by a significant activation of T cells and NK cells after 4 days of twice daily consumption (Mikolai et al., 2009).

A recent clinical study of patients with COVID-19 showed increased serum levels of GM-CSF (Zhou Y. et al., 2020). GM-CSF binds to its receptor on immune cells to stimulate the expression of cytokines. Therefore, few therapeutic approaches are suggesting to target GM-CSF to mitigate cytokine storm (Ayisha Sharma and Vaidya, 2020). In this background, withanolide A might be beneficial as it is found to have a substantial binding affinity with GM-CSF receptor in a molecular docking study (Posa et al., 2016).

The property of WS in optimizing immune response is being explored in the management of COVID-19. The Ministry of AYUSH, Govt. of India, has initiated multicentric clinical studies in India. These studies have been aimed to understand WS applications in the management of COVID-19 (CTRI/2020/05/025429).

### Cytokine Storm

Viral infections such as SARS-CoV-2 trigger excessive generation of pro-inflammatory cytokines known as cytokine storm (Tang et al., 2020). Cytokines mediate the cross-talk between immune cells and infected cells to clear infectious agents (Mehta et al., 2020). With this background, Table 1 shows the summary of the role of WS to reduce inflammatory cytokines.

**TABLE 1:** Suppression of inflammatory cytokines by withanolides and *Withania somnifera* extracts in several study models.

No	Intervention	Model	Cytokines	References
1	Withaferin A	Macrophages (RAW 264.7)	IL-1β, IL-6, IL-23, and TNF-α	Neog, Sultana, and Rasool (2018)
2	Withaferin A	Monocytes (THP-1)	IL-1α, IL-1β, IL-2, IL-3, IL-5, IL-10, IL-12p70, IL-18 B Pα, IL-13, IL-23, IL-33, IL-34, IP-10, GM-CSF, PDFG-AA, CCL2/mcp-1, CCL17/TARC, SDF1α/CXCL12, CCL20/mip-3α, KLK3, angiopoientin-1, IGFBP-2, TFF3, BAFF, BDNF, FLT3LG, IGFBP-3, ACRP30/adiponectin, GH, leptin, LIF, SHBG, aggrecan, angiogenin, HGF, NGAL, TSP-1, and CST3	Dubey et al. (2018)
3	Withaferin A	Murine bone marrow-derived macrophages	IL-6 and TNF-α	Noh et al. (2016)
4	Withaferin A	Murine bone marrow-derived dendritic cells	IL-1β	Kim et al. (2015)
5	WS root aqueous extract	Serum of collagen-induced arthritic rat	IL-1β, IL-6, and TNF-α	M. A. Khan et al. (2018)
6	Withaferin A	Synovial macrophages of arthritic rat	IL-1β, IL-6, MCP-1, TNF-α, and VEGF	Sultana, Neog, and Rasool (2017)
7	WS extract as dietary supplement	LPS and Con-A induced spleen lymphocytes of rats	TNF-α	Yamada et al. (2011)
8	Withaferin A	Lung tissues of mouse model of pulmonary fibrosis	IL-1β and TNF-α	Bale et al. (2018b)
9	WS root powder	Serum and ascitic fluid of pristane-induced mouse model of systemic lupus erythematosus	IL-6, NO, ROS, and TNF-α	Minhas et al. (2012)
10	WS root hydro-methanolic extract	Healthy mice	TNF-α	L Davis and Kuttan (1999)
11	WS root and leaves aqueous and ethanolic extract	Guinea pigs infected with *E. coli*	TNF-α	El-Boshy et al. (2013)
12	WS root ethanolic extract	PBMCs of healthy individuals and patients with rheumatoid arthritis	IL-1β, IL-12, and TNF-α	Singh D. et al. (2007)
13	WS root ethanolic extract	Macrophages (RAW 264.7)	NO	Singh D. et al. (2007)

The characterization of COVID-19 pathophysiology highlighted the decisive role of IL-1β and IL-6. It has been found that increased level of these cytokines is associated with the severe stage of COVID-19 (Aziz et al., 2020). WHO released an informal consultation to explore the potential role of available antagonists of IL-1β and IL-6 in the disease management. Tocilizumab, a monoclonal antibody and an IL-6 receptor inhibitor, is being considered to treat patients with COVID-19. This blueprint also stated the variability of IL-6 concentrations in patients with COVID-19. Therefore, it was recommended to restrict the tocilizumab treatment to patients with high IL-6 levels (“WHO R&D Blueprint COVID-19 Informal Consultation on the Potential Role of IL-6/IL-1 Antagonists in the Clinical Management of COVID 19 Infection” 2020) (Michot et al., 2020).

In such a scenario, WS may also be helpful to suppress IL-1β and IL-6 as evident in several models. WS aqueous extract along with fatty acids and withaferin A inhibited IL-6 and IL-1β released by monocytes, macrophages, and keratinocytes *in vitro* (Balkrishna et al., 2020) (Dubey et al., 2018) (Neog et al, 2018) (Sikandan et al., 2018). Withaferin A and WS aqueous extract significantly reduced serum levels of IL-1β and IL-6 secreted by bone marrow-derived dendritic cells and macrophages (Morsy et al., 2019) (Sultana etal., 2017) (Noh et al., 2016) (Kim et al., 2015) (Khan et al., 2018). Withaferin A also suppressed IL-1β expression of the fibrotic lung of a mouse (Bale et al., 2018a). WS root powder significantly reduced IL-6 secretion in ascitic fluid and serum of lupus erythematic mouse model (Minhas et al., 2012). Clinically, WS ethanolic extract attenuated IL-1β expression in PBMCs of healthy individuals and arthritic patients (Singh D. et al., 2007).

All these activities from several models demonstrate WS activity to suppress IL-1β and IL-6. Thus, WS can be considered for the investigation to mitigate cytokine storm in patients with COVID-19, with special reference to IL-1β and IL-6.

### Hyperinflammation

An aspect of hyperinflammation highlights COVID-19 as a chronic disease. It is characterized by alterations in cytokine milieu and inflammatory pathways (Tufan et al., 2020) (Gustine and Jones, 2020). The inflammatory pathways are mediated through several factors including cytokines, receptor proteins, inflammasomes, and nuclear factors, etc. Withanolides have been reported to regulate the inflammatory pathways such as NF-κB, JAK/STAT, Nrf2, and HIF-1. This favors withanolides in clinical application to manage chronic diseases associated with inflammation (White et al., 2016). The anti-inflammatory action of WS through suppression of inflammatory cytokines has been already shown in Table 1. This section highlights other mechanistic studies of WS that show interference in inflammatory pathways in the background of hyperinflammation in COVID-19.

Immune cells facilitate inflammatory signaling through several immune factors and receptor proteins. Peroxiredoxins (e.g., Prx1) expressed by alveolar macrophages are immune factors capable of inducing acute inflammation in the lungs (Knoops et al., 2016) (Kinnula et al., 2002) (Diet et al., 2007). Prx1 acts as DAMPs to bind TLR4 and stimulates the secretion of pro-inflammatory cytokines (Riddell et al., 2010). A piece of strong mechanistic evidence revealed that withaferin A inhibits the chaperone activity of Prx1 and reduces the infection-induced TLR4 expression in macrophages (Noh et al., 2016) (Zhao Q. et al., 2015). Furthermore, cytoplasmic glucocorticoid receptors (GRs) contribute to the anti-inflammatory effect upon binding of glucocorticoids (class of steroids) through distinct molecular mechanisms (Baschant and Tuckermann, 2010). Molecular docking of withanolide D targets 3D structural model of GPCR (expressed on inflammatory cells), which suggested curbing of inflammation (Sun and Ye, 2012) (Chinthakunta et al., 2018). Withaferin A showed the agonistic effect on GR with strong binding affinity comparable to that of fluticasone, a synthetic GR agonist. An anti-inflammatory effect was validated using *in vivo* analysis wherein withaferin A reduced granuloma (collection of macrophages that induces inflammation) in rats (Morsy et al., 2019).

Inflammasomes (intracellular multiprotein complexes) and macrophage polarization enables the regulation of inflammation in chronic diseases (Parisi et al., 2018) (Guo et al., 2015). AIM2 and NLRP3 inflammasomes represented by M1 macrophages and monocytes are responsible for the secretion of pro-inflammatory cytokines (Wang Z. et al., 2020). Withaferin A is found to modulate AIM2 inflammasome accompanied with a reduction in M1 macrophage-mediated pro-inflammatory cytokines and STAT3-induced macrophage repolarization (Neog et al., 2018) (Ngoungoure and Owona, 2019). Macrophage repolarization by withaferin A was also significant in arthritic rats, ameliorating the inflammatory response (Sultana et al., 2017). The severe cases of COVID-19 exhibited dysregulation of NLRP3 inflammasome, which is emerging as a potential therapeutic target (van den Berg and Te Velde, 2020) (Shah, 2020). Withaferin A also suppressed the expression of NLRP3 inflammasome in monocytes (Dubey et al., 2018), dendritic cells (Kim et al., 2015), and in lung tissues (H. M. Zhao et al., 2019). Additionally, administration of withaferin A normalized inflammation-induced FoxO3a gene expression in diseased (scleroderma) mouse model proportionate to that of a control group (Peng, 2010) (Bale et al., 2018b). A few oxidative enzymes such as lipoxygenases contribute to inflammation by inducing the production of leukotrienes (Rad̊mark and Samuelsson, 2009). An *in vitro* and *in silico* analysis of aqueous and hydroethanolic extracts of WS with 5-lipoxygenase (isolated from human polymorphonuclear leukocytes) showed a significant inhibition better than conventional anti-inflammatory agents (Madhusudan et al., 2016). WS aqueous extract also significantly reduced the lymphocyte proliferation in the inflammatory arthritic rat model (Rasool and Varalakshmi, 2006).

An activation of the NF-κB pathway is a decisive mechanism in inducing inflammation (Liu et al., 2017). It potentiates cytokine secretion and leukocyte recruitment to contribute to the inflammatory response. Therefore, it has been considered as a therapeutic target in inflammatory diseases (Lawrence, 2009). WS has emerged as a potential modulator of the NF-κB pathway with withaferin A as an inhibitor of NF-κB activation (Heyninck et al., 2014). Similarly, withaferin A also inhibited bacterial infection-induced NF-κB activation in macrophages and dendritic cells of mice (Noh et al., 2016). Withaferin A (Neog et al., 2018) (Bale et al., 2018a) and fatty acids of WS seeds (Balkrishna et al., 2020) reduced NF-κB expression in monocytes, macrophages, and fibrotic lung tissues of mice. WS aqueous extract was found to inhibit NF-κB activation in collagen-induced arthritic rats (Khan et al., 2018). The mechanistic studies revealed that NF-κB inhibition occurs due to the binding of withaferin A to the thiol group of cysteine residues (Heyninck et al., 2014) (Gambhir et al., 2015). Withaferin A found to block nuclear translocation of NF-κB in lymphocytes along with a significant reduction in cytokine release by monocytes (Gambhir et al., 2015) (Dubey et al., 2018). The ethanolic extract and withaferin A were found to inhibit nuclear translocation of NF-κB in PBMCs of healthy individuals and arthritic patients (Singh D. et al., 2007).

The role of WS in inflammatory diseases is explored by several researchers. Mazzio et al. explored several natural products for their anti-inflammatory activity in the context of the acute systemic inflammatory response to prevent sepsis. The study revealed explicitly that WS has better anti-inflammatory potential than all other natural products accompanied with negligible toxicity (Mazzio et al., 2016). Similarly, Minhas et al. investigated the prophylactic and therapeutic effects of WS in systemic lupus erythematosus. WS showed protective and anti-inflammatory effects before and after the disease induction, respectively. These effects were validated by attenuation of pro-inflammatory cytokines on the administration of WS root powder (Minhas et al., 2011) (Minhas et al., 2012). A rectal application of WS extract gel formulation also exhibited remedial effect against inflammatory bowel disease to combat inflammation (Pawar et al., 2011).

The collected mechanistic studies suggest that WS and withanolides restrict inflammatory response through regulating cytokine expression, modulating inflammatory receptor proteins, and inhibiting the NF-κB pathway. This encourages the investigation of WS in COVID-19 hyperinflammation.

### Vascular Integrity and Alveolar Consolidation

The effect of inflammatory mediators on blood vessels that are carrying them is obvious. Patients with COVID-19 showed that immune cells, inflammatory cytokines, and vasoactive molecules increase gaps between endothelial cells that line blood vessels. This leads to vascular leakage that causes infiltration of inflammatory cells (Teuwen et al., 2020). As a result, immune cells and cytokines enter previously occupied alveolar space, leading to consolidation that is visible through lung imaging (Jacobi et al., 2020).

Withaferin A significantly suppressed apoptosis of endothelial cells to pacify disruption of the blood–brain barrier in a mouse model of traumatic brain injury (Zhou Z. et al., 2020). An individual vascular permeability study revealed that withaferin A protects endothelial barrier *in vitro* and *in vivo*. Withaferin A significantly inhibited HMGB1-mediated barrier disruption, acetic acid-induced hyperpermeability, and restricted transendothelial migration of leukocytes (Lee et al., 2012) (Ahmad and Dar, 2017). WS aqueous extract may prevent histamine-mediated contraction of endothelial cells, which leads to avoid venular intercellular gaps (Joris et al., 1972) (Sahni and Srivastava, 1993).

Patients with COVID-19 showed elevated levels of vascular endothelial growth factor (VEGF), a vascular permeability inducing factor, in the blood (Senger et al., 1993) (Huang et al., 2020). A clinical trial (NCT04275414) to explore bevacizumab, an anti-VEGF, in patients with COVID-19 has been registered with clear pathological rationale (“Bevacizumab in Severe or Critical Patients With COVID-19 Pneumonia (BEST-CP)” 2020). Molecular docking studies revealed that withaferin A is a potent inhibitor of VEGF (Saha et al., 2013). Withaferin A suppressed VEGF expression in macrophages of arthritic rats (Sultana et al., 2017) and lung tissues of pulmonary fibrotic mice (Bale et al., 2018b).

These studies support the protective and preventive effects of WS in vascular permeability-induced alveolar consolidation. Thereby, WS may help reduce breathlessness in patients with COVID-19. Therefore, systematic clinical studies are required to validate this effect of WS.

### Inflammation-Induced Organ Failure

The clinical complications of COVID-19 have been demonstrated to influence multiple vital organs (Zheng K. I. et al., 2020). The systemic inflammatory response coupled with direct viral association with several organs through the ACE2 receptor amplifies the complications. Multi-organ failure including liver damage, renal failure, and cardiovascular impairment contributes to death (Zaim et al., 2020). The systemic inflammation is a plausible cause of consecutive organ failure. The inflammatory mediators released by immune cells circulate via blood and induce inflammation of several organs leading to damage (Cao, 2020).

The clinically evident holistic approach of Ayurveda designates health as the central point of concern. Ayurveda interventions are aimed at strengthening homeostatic mechanisms and optimizing adaptation limits. Unlike the organ-specific approach of modern biomedicine, Ayurveda focuses on systemic revitalization to protect organs thereby avoiding failure (Payyappallimana and Venkatasubramanian, 2016). WS has emerged as a widely acclaimed organ-protective drug capable of potentiating several organs to fight against infections and inflammation (Baliga et al., 2015). This section of the review highlights the probable role of WS in the biological consequences of inflammation-induced organ failure in COVID-19.

#### Brain

The neurological deformations are associated with COVID-19 pathophysiology. The major clinical observations of patients incorporate encephalitis, necrotic hemorrhage, and epileptic seizures (Carod-Artal, 2020). A systematic review of neurological manifestations in COVID-19 describes headache and anosmia as the most common symptoms (Whittaker et al., 2020).

Neuroinflammation triggered by immune cells is a key mechanism to induce neurological complications (Vito et al., 2017) (Vito et al., 2017). WS leaf aqueous extract ameliorated inflammatory cytokine-induced neuroinflammation by inhibiting the NF-κB pathway in the rat model (Gupta and Kaur, 2018). The same extract also showed a neuroprotective effect by normalizing MAP2 expression in microglial cells (Gupta and Kaur, 2019). Therefore, this extract can be a promising candidate to prevent neuroinflammation. Withanolide A significantly inhibited cerebral ischemia-induced apoptosis and necrotic cell death, which emphasizes its ability to maintain neural network and cognitive function (Mukherjee et al., 2020) (Kuboyama et al., 2005). Withanolide A also found to prevent hypoxia-induced neurodegeneration through modulation of glutathione biosynthesis (Baitharu et al., 2014). WS hydroalcoholic extract is also reported to be an anti-stress agent in an animal model of depression (Tripathi et al., 1998). Several withanolides can cross the blood–brain barrier, which promote their usage in developing a therapeutic and preventive drug for neurological disorders (Vareed et al., 2014).

#### Heart

The COVID-19 patients also showed heart-related complications such as cardiomyopathy and myocarditis. The pathological observations revealed elevated levels of cardiac troponin I, IL-6, and ACE2 receptors (Adão and Guzik, 2020).

Myocarditis and further heart dysfunction are coupled with systemic inflammation. The alcoholic extract of WS leaf normalized troponin I release in the blood and thereby preserved structural and functional integrity of contractile myocardium in a rat model (Khalil et al., 2015). WS root powder has shown strong inhibition of proinflammatory cytokine IL-6 in a mouse model of systemic inflammation (Ingawale et al., 2020). These evidences suggest the probable cardioprotective effect of WS in patients with COVID-19.

#### Liver

Patients with COVID-19 with severe categories showed liver damage, which was evident by abnormal ranges of liver enzymes and other markers. There was a significant rate of mortality due to liver failure on SARS-CoV-2 infection (Bangash et al., 2020). Hepatic inflammation can also be considered as a cause of damage.

Withanolide-rich fraction isolated from WS root methanolic extract found to restore the marker enzyme levels in drug-induced hepatic cytotoxicity in rat models of cytokines (Devkar et al., 2016). Pre-administration of withaferin A also showed the hepatoprotective effect on bromobenzene-induced injury in a mouse model evidenced by normalized functional enzymes (Vedi and Sabina, 2016). WS root powder normalized carbendazim-induced histopathological alterations in rat liver (Akbarsha et al., 2000).

#### Lungs

The nasal tract, nasopharyngeal cavity, and lungs are the main sites for SARS-CoV-2 infection. Pulmonary inflammation, anosmia, respiratory distress, and lung endothelial dysfunction with endothelitis are the major symptoms associated with COVID-19 (Gengler et al., 2020) (Varga et al., 2020). Reports also highlighted that patients with pulmonary fibrosis are more prone to SARS-CoV-2 infection (George et al., 2020).

Endothelial dysfunction due to inflammation results in pulmonary hypertension. WS root powder attenuated endothelitis in a rat model by limiting expression of proliferating cell nuclear antigen (PCNA). It also suppressed the level of inflammatory markers IL-10 and TNF-α by regulating the NF-κB transcription factor (Kaur et al., 2015b), while polysaccharide arabinogalactan from WS has a distinct antitussive activity through the action of the mu-opioid receptor pathways (Nosálová et al., 2015). Withaferin A from WS has shown protective effects against pulmonary fibrosis and lung damage by suppressing the expression of growth factor b1 in animal studies (H. M. Zhao et al., 2019).

Anosmia is neuroinflammatory dysfunction of olfactory cells due to entry of COVID-19 through the nasal tract. WS has a neuroprotective activity by regulating Sema3a factor in olfactory cells in a mouse model (Raghavan and Shah, 2015).

#### Gastrointestinal Tract

GI tract dysfunction is also found to be associated with COVID-19. A population study of COVID-19 patients reported common symptoms as nausea, diarrhea, abdominal pain, anorexia, and vomiting (Yang and Tu, 2020). Pathological findings indicated a high quantity of ACE2 expression in proximal and distal enterocytes of the small intestine (Tian et al., 2020).

The immune response-induced inflammation against COVID-19 is the main cause of GI tract dysfunction. WS root aqueous extract expressed antidiarrheal activity and maintained gastrointestinal mobility in the rat model by inhibiting cyclooxygenase and regulating inflammatory markers induced by NF-κB transcription factor (Pawar et al., 2011). Withaferin A from WS also has shown anti-inflammatory potential in the pancreas and liver by upregulating Nrf2 transcription factor and restoring antioxidant mechanism in experimental mice (Tiruveedi et al., 2018).

#### Kidney

The renal system is also found to be a target of COVID-19. Pathological findings reported direct entry of COVID-19 in kidney cells through increased ACE-2 receptor, while the inflammatory response to infection shows proteinuria and increased levels of serum creatinine and urea nitrogen (Valizadeh et al., 2020).

The inflammatory mechanism is responsible for renal dysfunction. WS root powder was found to show a nephroprotective effect by stabilizing urea nitrogen and creatine level and restoring antioxidant enzyme expression in animal models (Jeyanthi and Subramanian, 2010) (Vedi et al., 2014). This is followed by normalizing histopathological changes in the kidney (Vasavan et al., 2020). Withaferin A balances apoptotic markers to prevent cytotoxicity of bromobenzene in the kidney cells (Vedi and Sabina, 2016).

#### Muscles

Rhabdomyolysis, myalgia, and muscle weakness are symptoms found in COVID-19 patients (De Giorgio et al., 2020). They persist after recovery from viral infection as a post-COVID-19 complication (Huang et al., 2021).

Several clinical trials involving the administration of WS aqueous extract found an increase in the overall muscle strength (Wankhede et al., 2015) (Ziegenfuss et al., 2018). Rhabdomyolysis is associated with altered levels of serum urea and creatinine (Walid, 2008). The WS root extract also found to reduce elevated levels of serum urea and creatinine in rats (Shimmi et al., 1970).

#### Pancreas

The complications of COVID-19 have also found to induce acute pancreatitis (de-Madaria and Capurso, 2021). The biochemical reports of COVID-19 patients revealed increased levels of pancreatic enzymes including lipase and amylase (de-Madaria et al., 2020).

Withaferin A (Tiruveedi et al., 2018) and aqueous extract of WS roots (Anwer et al., 2012) showed protective effects against acute pancreatitis through enzymatic modulation of oxidative stress and inflammation in animal model. Additionally, withaferin A also reduced the serum levels of lipase and amylase enzymes elevated in a mouse model of acute pancreatitis (Tiruveedi et al., 2018).

#### Gonads

COVID-19 is associated with gonadal inflammation. Primary pathological symptoms include damage to spermatozoan, abnormal secretion of sex hormones, and increased level of ACE-2 in Leydig cells in testes (Li R. et al., 2020).

Inflammation-induced cytokines are the main molecules for the dysfunction of the testes. Administration of methanolic extract of WS root has shown regulation of gonadotropic hormone by controlling GABA neurons in a mouse model (Bhattarai et al., 2010). This same extract also restored prolactin and estrogen level by inhibiting aromatase in a fish model (Nasimi Doost Azgomi et al., 2018).

## Possible Role of *Withania somnifera* in COVID-19 Management

An intervention provided in addition to mainstream administration is considered as an adjuvant. The primary aim of the adjuvant is to enhance the beneficial effects and reduce the adverse effects of mainstream administration. This section of the review discusses the potential of WS as an adjuvant to current pharmacotherapeutics and vaccine development in COVID-19.

### Therapeutic Adjuvant

The current therapeutic approaches in the management of COVID-19 involves the use of hydroxychloroquine, chloroquine, azithromycin, and emergency antiviral drug remdesivir (“Revised Advisory on the Use of Hydroxychloroquine (HCQ) as Prophylaxis for SARS-CoV-2 Infection” 2020) (“Clinical Management Protocol” 2020). The beneficial effects of these drugs in COVID-19 are being explored extensively through several clinical trials. The rationale behind using these drugs is empirical, and the clinical data for use in COVID-19 are inconclusive. However, wide use of these drugs is premature and might expose patients to rare but serious harms (Ferner et al., 2020).

The major concern with the use of hydroxychloroquine and chloroquine is cardiac arrhythmia (Borba et al., 2020) (Blignaut et al., 2019). A combination with azithromycin was found to worsen this condition by increasing the risk of cardiovascular mortality, angina, and heart failure (Lane et al., 2020). The arrhythmic complication was also observed in patients with COVID-19 treated with hydroxychloroquine and azithromycin (Chorin et al., 2020). Similarly, remdesivir was found to deteriorate the condition of COVID-19 patients by triggering cardiopulmonary (5%) failure and respiratory failure or ARDS (10%) (Wang Y. et al., 2020). In this background, WS is reported to show cardiorespiratory protection. The pharmacological effects of WS in several cardiovascular diseases have been explored (Ojha and Arya, 2009). It was found to augment endogenous myocardial antioxidant enzymes (Mohanty et al., 2008) and restore altered hemodynamic parameters (Mohanty et al., 2004). An exploration of WS in a clinical trial was found to improve cardiorespiratory endurance (Shenoy et al., 2012).

A few cases reported hydroxychloroquine-associated hepatic failure leading to death. The histopathological observations showed necrosis of liver parenchymal cells and moderately percolated inflammatory cells (Makin et al., 1994). WS is also a hepatoprotective agent capable of normalizing drug-induced altered liver enzymes (Al-Awthan et al., 2014). Withanolides were found to reduce inflammatory mediators of rat liver induced by acetaminophen overdose. The histological alterations such as liver necrosis were also restored by withanolides in a dose-dependent manner (Devkar et al., 2016). Moreover, WS is a safe drug and there have been no observations of hepatotoxicity by WS in clinical trials (Paramadhas and Alagirisamy, 2016).

An approach of convalescent plasma therapy is also showing promising results in COVID-19 management. The beneficial effects of convalescent plasma therapy have been demonstrated by increased neutralizing antibody titer accompanied by reduced SARS-CoV-2 RNA in patients (Rajendran et al., 2020). WS is also investigated for its effects on humoral immune response. WS root powder and extract significantly increased circulating antibody titer in healthy and immunocompromised mice (Davis and Kuttan, 2000) (Bani et al., 2006). WS extract is also capable of enhancing antibody-dependent cellular cytotoxicity wherein circulating antibody recognizes infected target cell and recruits NK cells to induce apoptosis (Davis and Kuttan, 2002). A hydroalcoholic extract of WS augmented IgG and IgM titers to reach peak value and serum levels of IgG2a over IgG1in ship RBC immunized mice (Malik et al., 2007). 2,3-Dihydro-3-sulfonile withanone isolated from WS leaf extract also triggered IgG2a secretion by LPS-induced splenocytes (Khan S. et al., 2009). Interestingly, the expression of IgG2a is correlated with the clearance of influenza virus (Huber et al., 2006). This suggests a probable protective effect of WS against SARS-CoV-2 through antibody regulation.

Overall, the preceding studies suggest that WS may show benefits to decrease adverse effects of drugs and adds up to antibody response in COVID-19 management. The multimodal activities of WS described in the previous section may exhibit the probable synergistic effects. Therefore, WS can be a promising candidate to be considered as a therapeutic adjuvant in COVID-19 management.

### Vaccine Adjuvant

The COVID-19 pandemic has accelerated vaccine development. Several research institutes and industries have paced up the vaccine studies. Amid the rapidly spreading virus, there is an urgent need for an effective vaccine (Kaur and Gupta, 2020). Our team had previously proposed a combination of vaccines and herbal immunostimulants as an innovative approach to increase vaccine efficacy (Gautam et al., 2008). Gautam et al. have explored the potential of WS as a vaccine adjuvant to boost vaccine immunogenicity. Oral feeding of WS aqueous extract to DPT vaccine-immunized mice significantly increased antibody titer on challenge with live *B. pertussis* cells. This was accompanied by improved overall health status and reduced mortality (Gautam et al., 2004). Our team also patented an innovative method to obtain withanolide-rich WS fraction in the context of vaccine adjuvant (Jadhav et al., 2010).

In the background of viral diseases, the immune responses to influenza and SARS-CoV-2 infection were found to have similar aspects (Zheng and Perlman, 2018). A recent study demonstrated the key role of NK cells in an adaptive immune response against influenza infection (Mooney et al., 2020). WS is known to enhance NK cell activity of cell lysis and antibody-dependent cellular cytotoxicity in healthy mice (Davis and Kuttan, 2002). Considering all the previously mentioned leads, it is indicative to explore WS in COVID-19 vaccine development as an adjuvant.

## Probable Role of *Withania somnifera* in the Clinical Management of COVID-19

The multimodal effects of WS discussed in this review point toward its use in the clinical management of COVID-19. Depending upon the clinical observations, COVID-19 has been classified into three levels of severity such as mild, moderate, and severe (“Clinical Management Protocol”, 2020) (Siddiqi and Mehra, 2020). This section highlights the probable effects of WS in the background of the severity of COVID-19.

### Mild

An early infection phase with primary nonspecific symptoms such as malaise, fever, and dry cough is considered as a mild level. The treatment strategies involve the use of antipyretic and antiviral drugs with a focus on symptomatic relief (“Clinical Management Protocol” 2020) (Siddiqi and Mehra, 2020). WS is prescribed in Ayurveda practice to manage these primary symptoms. The fever is mediated by COX-2 enzyme and prostaglandin molecules (Li et al., 2001) (Kita et al., 2015). The antipyretic effect of WS is evident through COX-2 inhibition (Yu and Kim, 2013) (Devkar et al., 2016) (Prabhakaran et al., 2012) and prostaglandin suppression (Min et al., 2011). WS needs to be investigated for its efficacy in viral fever and other symptoms. The antiviral properties of WS mentioned in this review seem promising and need to be tested clinically in patients with mild symptoms.

### Moderate

The second moderate level considers pulmonary involvement with or without hypoxia. This stage is characterized by the recruitment of immune cells at the infected site, i.e., alveoli followed by pulmonary inflammation, alveolar consolidation, and vascular permeability. At this stage, a rational administration of anti-inflammatory therapy such as methylprednisolone is suggested (“Clinical Management Protocol” 2020) (Siddiqi and Mehra, 2020). WS is well known for its anti-inflammatory action in several inflammatory diseases. It normalizes the inflammatory signals to restore normal physiological functioning of immune cells in disease conditions (Gupta and Singh 2014) (Khan et al., 2019) (Kaur et al., 2015a). Some COVID-19 patients experience a secondary bacterial infection. WS may act as a drug of choice in such cases (“Clinical Management Protocol” 2020) (Rawat and Bisht, 2014) (Owais et al., 2005). However, this activity needs to be tested in the COVID-19 scenario. The multimodal effects of WS may be useful for the speedy recovery of patients and may prevent from progressing to a severe disease.

### Severe

A severe stage of systemic hyperinflammation is caused by immune cells-induced cytokine storm. The dysfunction of cell-mediated immunity due to decreased T cell count and increased secretion of inflammatory mediators is observed in the severe stage (Siddiqi and Mehra, 2020). An immunomodulatory intervention is recommended for physiological management of the severe condition. WS is a promising immunomodulatory agent to maintain physiological immune harmonizing. It helps in balancing Th1/Th2 response to stimulate cell-mediated immunity in mice (Khan B. et al., 2006) (Malik et al., 2007) (Kour et al., 2009) (Khan S. et al., 2009) (Bani et al., 2006) (Susheela Kushwaha et al., 2012a). Furthermore, the antibodies produced by the humoral response of WS may be beneficial (Malik et al., 2007) (Khan S. et al., 2009) (Bani et al., 2006) (Davis and Kuttan, 2000). The systemic inflammation may lead to multi-organ failure. The reported anti-inflammatory and organ-protective effect of WS (Tiwari et al., 2014) may be useful in reducing the severity of inflammation-induced organ damage. As discussed above, WS may be beneficial for patients at different stages of the infection and may prevent progression to the severe stage.

The post-discharge detection of viral titer in recovered patients is another major issue. There is a significantly noticeable number of relapse cases in recovered patients. However, the mechanism of relapse remains unknown (An et al., 2020). The patients are recovering based on their physiological resistance when subjected to empirical treatments (Shin, 2020). Therefore, there is a need for a relatively safe prophylactic agent capable of sustaining physiological resistance through immune balance. WS can be a promising option to execute prophylaxis against relapse. It is a relatively safe drug even for long-term administration (Raut et al., 2012). Moreover, pre-existing comorbidities add up to the complications. Patients with COVID-19 suffering from comorbidities often need to undergo disease-specific poly-pharmacy regimens. WS being a safe drug can be used as one of the complementary medicines to mitigate COVID-19 symptoms. The adaptogenic effects of WS might re-establish physiological balance in the background of the stressed conditions of comorbidities (Singh et al., 1982).

The clinical safety profile of WS aqueous extract has already been studied. A dosage of 625 mg twice a day was well tolerated by almost all the healthy volunteers without any toxicities. Organ function tests were found normal even after a month of administration (Raut et al., 2012). A systematic review also advocated the WS root to be safe and clinically effective in several disorders including schizophrenia, rheumatoid arthritis, diabetes, and infertility. It was also found safe in preclinical toxicity studies carried out for 8 months (Tandon and Yadav, 2020). However, systematic toxicity studies on WS are needed in the COVID-19 scenario.

## Discussion

The experimental trends generated from healthy and disease models indicate that WS has a potential for 1) maintaining immune homeostasis, 2) regulating inflammation, 3) suppressing pro-inflammatory cytokines, 4) organ protection (in the nervous system, heart, lung, liver, and kidney), and 5) anti-stress, antihypertensive, and antidiabetic activities. The dose of WS extracts ranged from 10–300 mg/kg in various preclinical experimental models have been discussed in this review. However, it was found that the concentration range of 50–200 mg/kg of different extracts of WS and 2 mg/kg of withaferin A tested in animal models significantly showed anti-inflammatory, cytokine regulatory, and immunomodulatory effects. Moreover, 10–50 μg/ml of withaferin A stimulated cytokine expression in monocyte and macrophage cell lines (Supplementary Material provides details on the type of extract/metabolite, experimental model, activity, and dose range).

The possible role of WS in regulating various biochemical processes in COVID-19 pathophysiology can be extrapolated from reported studies. The current understanding of the pharmacotherapeutic treatment of COVID-19 relies on immunological phenomena associated with viral infections. It has been proposed that the therapeutic strategies to improve immune profile could be beneficial in clinical outcomes. For example, an antiviral drug remdesivir with the immunomodulator tocilizumab has been recognized to refine pharmacological efficacy (Michot et al., 2020). In such a scenario, WS being a potent immunomodulator can be considered as an excellent alternative given its proven safety and efficacy profile.

The next phase of COVID-19 vaccine development would be an association with synthetic adjuvant. WS, having proven attributes such as affordability, efficacy, and safety, can be evaluated as a vaccine adjuvant. Systematically and ethically designed studies are the need of the hour. These studies with rational measurable outcomes (e.g., antibody titer, and cytokine profile, etc.) would throw light on the fundamental benefits of WS.

The post-COVID-19 complications also deserve a serious attention to achieve quality of life (Cortinovis et al., 2021). Several clinical follow-up studies on recovered COVID-19 patients reported persisted fatigue, muscle weakness, and organ dysfunction (Huang et al., 2021) (Zhao et al., 2020). This warrants implication of muscle-strengthening (Ziegenfuss et al., 2018) and organ-protective (Kaur et al., 2015b) action of WS.

The study of literature suggests that WS has important molecular and pharmacological characteristics to act as a therapeutic adjuvant for prophylaxis and treatment of COVID-19. Therefore, WS may synergistically improve the clinical outcomes of COVID-19 pharmacotherapeutics in current use. WS may be explored in healthcare workers undergoing COVID-19 vaccination for modulation in antibody titer to understand the real-world adjuvant potential. Besides, WS may have a salutary effect on other comorbidities associated with the COVID-19 disease. Our recently published review has addressed this possibility in cancer (Saggam et al., 2020). To realize the potential of WS in COVID-19, additional evidence needs to be generated by experimental validation of *in silico* observations followed by clinical studies. The focus on evaluating pharmacokinetic–pharmacodynamic profile and drug interactions along with safety and efficacy in healthy volunteers and patients with COVID-19 for prophylactic and therapeutic use is required. This review attempts mapping of phytochemical diversity and reported pharmacological activities of WS with COVID-19 pathophysiology. The review proposes potential pharmacological activities of WS extracts and metabolites in the context of COVID-19, which are based on rational extrapolations from available literature leading to testable hypotheses. However, there is a need for systematic studies with appropriate controls to validate these hypotheses in suitable experimental models. Moreover, the activity of any botanical depends on a number of variables including geographical variation of species, cultivation and collection methods, extraction process, and administration (route, dose, and duration). The extract should be prepared from the authentic and standardized raw material with a robust protocol to ensure batch-to-batch reproducibility, potency, safety, and efficacy. These sources of variability need to be accounted during experimental designs for exploring WS effects.

The multidimensional research on WS with a deeper understanding of biological mechanisms is a promising area for future exploration as a therapeutic adjuvant. Given the recent observations that COVID-19 pandemic will have a long-term trajectory, there will be a significant need for research and development of COVID-19 therapeutics and adjuvants (Lurie et al., 2021). In this context, the current research synthesis about WS indicates a potential role in COVID-19 management and may serve as a rational strategy for exploration of other *Rasayana* botanicals.
